# 健康是本钱: health is my capital: a qualitative study of access to healthcare by Chinese migrants in Singapore

**DOI:** 10.1186/s12939-017-0567-1

**Published:** 2017-06-15

**Authors:** Wai Jia Tam, Wei Leong Goh, Jeffrey Chua, Helena Legido-Quigley

**Affiliations:** 1 Saw Swee Hock School of Public Health, National University of Singapore, Singapore, Singapore; 2 HealthServe Pte Ltd, Singapore, Singapore; 3 London School of Hygiene and Tropical Medicine, London, UK

**Keywords:** Singapore, China, Migrants, Healthcare access

## Abstract

**Background:**

Since the 1970s, Singapore has turned into one of the major receiving countries of foreign workers in Southeast Asia. Over the years, challenges surrounding access to healthcare by Chinese migrant workers have surfaced globally. This study aims to explore the experiences of Chinese migrants accessing primary and secondary/tertiary healthcare in Singapore, and the opportunities for overcoming these barriers.

**Methods:**

We conducted 25 in-depth interviews of 20 Chinese migrants and five staff from HealthServe, a non-governmental organization serving Chinese migrants in Singapore from October 2015 to January 2016. Interviews were transcribed and analysed inductively adopting thematic analysis.

**Results:**

Chinese migrants in Singapore who were interviewed are mainly middle-aged breadwinners with multiple dependents. Their concept of health is encapsulated in a Chinese proverb “健康是本钱”, meaning “health is my capital”. Health is defined by them as a personal asset, needed to provide for their families. From their health-seeking behaviors, six pathways were identified, highlighting different routes chosen and resulting outcomes depending on whether their illness was perceived as major or minor, and if they sought help from the private or public sector private or public sector. Key barriers were identified relating to vulnerabilities during the migration process, during their illness, when consulting with healthcare providers, and during repatriation. A transactional doctor-patient culture in China contrasts with the trust migrants place in Singaporean’s public health system, perceived as equitable and personable. However, challenges remain for injured migrants who sought help from the private sector and those with chronic diseases.

**Conclusions:**

Policy recommendations to increase patient autonomy enabling choice of healthcare provider and provide for non-work related illnesses are suggested. Partnerships between migrant advocacy organizations and various stakeholders such as hospitals, government agencies and employers can be strengthened.

## Background

Since the 1970s, Singapore has turned into one of the major receiving countries of foreign workers in Southeast Asia [1]. In December 2014, approximately 1,355,700 workers were foreigners, constituting about 30% of the workforce, of which 322,700 were construction workers [2]. Singapore is currently the second largest global market for Chinese labour [1, 3]. An estimated 200,000 mainland Chinese workers in Singapore are employed in construction sites, factories, shops and restaurants [3].

Over the years, challenges surrounding access to healthcare of Chinese migrant workers have surfaced globally [4–7]. Like in many parts of the world, migrant workers in Singapore face high-risk occupational hazards, food insecurity and poor working conditions [8–10]. In Singapore, the first cluster of locally-transmitted Zika virus infection was reported on 27 Aug 2016 after our study was conducted, involving a large number of migrant workers in the construction industry [11]. Since 2007, migrant workers in Singapore were no longer eligible for state medical insurance and subsidized health care, which is known to be more affordable than private healthcare [4, 12]. According the Ministry of Manpower (MOM) regulations under the Employment Act (Chapter 91A), employers are responsible for any necessary medical healthcare of their employed migrant workers, whether or not the condition is work-related [13]. Employers are required by law to purchase and maintain medical coverage of at least $15,000 per year for the worker’s inpatient care and day surgery [14] and to provide work injury compensation insurance, including claims for medical expenses, permanent incapacity and death [15]. Migrant workers are also entitled to their gross rate of pay by their employers, if they are on paid hospitalization or outpatient sick leave [16].

Nonetheless, studies and the media in Singapore have demonstrated the challenges faced by migrant workers, in spite of these laws [17, 18]. For example, a local study exploring the health utilization patterns of 525 migrant workers from India, Bangladesh and Myanmar in Singapore revealed that the majority of migrant workers could access care, but certain groups faced challenges in prompt access, due to inadequate knowledge about healthcare insurance plans [4]. Another study on Chinese migrants in Singapore revealed the cultural challenges in the tense hierarchy system within Chinese construction work communities [9]. However, no recent studies have addressed the strengths and challenges faced by Chinese migrant workers in accessing healthcare in Singapore, amidst a rapidly evolving healthcare landscape. Challenges which arise in a culturally diverse society like Singapore can cause a disconnect between the dominant culture expressed through healthcare systems and that of minority groups, and should be explored [19]. This study thus aims to address this gap by exploring the experiences of Chinese migrants accessing primary and secondary/tertiary healthcare in Singapore, and the opportunities for overcoming these barriers.

## Methods

### Study design and sampling

A qualitative study design was chosen. In-depth interviews were conducted with 20 Chinese migrants and five full-time staff or part-time volunteers at HealthServe from October 2015 to January 2016. HealthServe was selected as the study site as it is a non-governmental organization (NGO) providing support for vulnerable migrant workers from China, Bangladesh, India, Myanmar, Thailand and Sri Lanka [20].

Table 1 shows age range and country of origin for all migrant participants.

**Table 1:** Migrant participants’ age range and province of origin

Province of origin	Age 31–40 years	Age 41–50 years	Age 51–60 years
Jiang Su	3	7	1
Hebei	2	1	1
Henan	0	2	0
Shandong	0	1	0
Sichuan	0	1	0
Anhui	0	1	0
Total	5	13	2

Mainland Chinese migrants utilizing services provided by HealthServe and its staff and volunteers were purposively sampled.

Chinese migrants were existing or previous outpatients. All were male and above the age of 21, as only male manual labourers are hired in Singapore. Their length of stay in Singapore varied between 2 months to 15 years. Staff and volunteer interviewees had at least 1 year full-time working or 2 years volunteering experience with migrants respectively.

Each interview on average lasted 90 min. Interviews with migrants were conducted in Mandarin, while interviews with staff or volunteers were conducted in English. All recordings were transcribed directly into English. Excerpts of transcribed text where quality checked throughout the process. Migrant participants were invited to share their journey of migration from China to Singapore as well as their healthcare experiences once they had moved to Singapore (Table 2). The interviews were conducted mostly by TWJ at HealthServe’s premises. Interviewees were compensated for their time with a token of appreciation worth USD5. Identifiers numbering 001 to 025 were given to each participant to protect their identities, with “M” referring to migrants, and “S” referring to staff or volunteers from HealthServe.

**Table 2:** Questions posed to migrant participants

Topic	Questions
Background	1. Could you tell me about yourself and your family?
Migration and Adjustment	2. Could you share with me about your decision to move to Singapore? 3. How has your journey been in moving from China to Singapore? 4. What were some of the challenges you faced in moving to Singapore? 5. What are your working hours and conditions like? 6. How is your relationship with your employer and colleagues?
Healthcare in Singapore	7. What does being healthy mean to you? 8. How has your experience been in seeking healthcare support and treatment here in Singapore? (prove for availability, affordability, acceptability, appropriateness) 9. Would you like to share any experiences you have encountered in the healthcare system? 10. What are some strengths and weaknesses of the healthcare system and healthcare providers you have encountered in Singapore? 11. What are some of the cultural barriers you’ve faced in accessing healthcare in Singapore? 12. How do you think healthcare providers in Singapore can be culturally more sensitive to you and your needs here in their delivery of healthcare? 13. How has your experience in HealthServe been?
Recovery and Repatriation	14. What are your thoughts about recovery and your future plans?

### Analysis

This study followed an interpretativist approach where interviews are believed to provide access to how participants understand and perceive their own reality and how they chose to report it. Our aim was to explore participants’ experiences in the migration process and in their access to primary, secondary and tertiary healthcare services. We framed their responses based on their accounts rather than within existing frameworks to analyse access to health care from the health services and/or health systems literature [21–23], although Penchansky and Tomas’ [21] and Levesque’s et al. [23] were used to inform the design of the interview topic guide. In this paper we conceptualise “access to healthcare” as the opportunity to have healthcare needs fulfilled at the time these are needed [23, 24]. Moreover, the dimensions used by Penchansky and Tomas’ [21] and Levesque’s et al. [23] to define access (i.e. availability, affordability, acceptability, appropriateness) are referred to throughout the paper and particularly when presenting the different pathways to healthcare accessed by Chinese migrants in Singapore.

Thematic analysis was used to identify data themes primarily inductively. NVivo 10 was used to code and organize data. Categories and themes were not decided prior to coding, but induced from the data itself. Notes on each line of transcripts were organized into major categories. Next, selective coding was used once major themes had emerged. This involved repeatedly verifying themes after rechecking transcribed data. Coding finished when analysis produced no new codes and all data were accounted for in core themes. Throughout the analysis authors identified opinions that fit the majority and deviant cases that produced exceptions. Thematic saturation was reached when there were fewer surprises in the data and no more new codes, themes and patterns emerged.

### Ethics

Given the sensitivity of the topic, researchers emphasized that participation was anonymous and confidential and participants could withdraw from the interview at any time. An ethical dilemma which arose was when participants approached the interviewer for personal help in their medico-legal issues. They were directed back to HealthServe for assistance. An information sheet, outlining study aims, confidentiality, anonymity, and audio recording was provided and explained to all participants. Written informed consent was obtained. All audio recordings and transcripts were linked by pseudonyms to ensure anonymity and stored securely in password-protected files to ensure confidentiality. Ethics approval was provided by the Institutional Review Board of the National University of Singapore.

## Results

Findings were categorized under three themes and six subthemes. We organized the results following the journey of migration: beginning from the point of migration, adjusting to a foreign work culture, experiencing in healthcare access overseas, recovering from illness and ending with plans of repatriation. Migration experiences include the reasons for migration and key information sources. Accessing healthcare focuses on migrants’ perspective of health, and their journeys through the public and private health sectors for major and minor illnesses, from which we identified pathways of access. Recovery and repatriation include the experience of waiting for their case to be settled and plans to return to China. The barriers which migrants faced through their healthcare journey were consolidated in a figure highlighting different points of vulnerability, from their existing vulnerabilities during the migration process, through their point of injury or illness and healthcare consultation, to the point of discharge, recovery and repatriation. A participant’s story brings together the factors affecting the Chinese migrant healthcare experience in Singapore, based on the pathways identified and highlighting points of vulnerability.

### Migration experience of mainland chinese migrants

#### Main reasons for migrating: finance and family support

All migrant participants reported finance as a top reason for migration. This was closely linked to the need to support their families, comprising elderly dependants and young children. Their profiles were typically middle-aged breadwinners who worked in agriculture, construction or odd-jobs. Those with sons had the added financial burden of buying them houses for their betrothal.

Major reasons for choosing Singapore as a destination included higher pay, security, and the presence of a mandarin-speaking community. Several migrants expressed the need to find a politically stable country which would provide steady work.

### “*Laoxiang*” (Village Friends) and “*Zhongjie*” (Agents) are key information sources

Main information sources for migration overseas included “*laoxiang*” (village friends), “*zhongjie*” (agents), and occasionally, the Internet. Nearly all migrants mentioned knowing friends or family who had ventured abroad and thus wanted to follow suit, based on the assumption that their ventures were fruitful.

*I see people from my hometown going to Singapore every year. So I thought things must be good overseas. (M001)*

Some went abroad alone, while many, with friends. Experiences through word-of-mouth from trusted friends were highly valued. Agents, who collect a fee between USD$3000 to $5000, match workers to companies abroad, also had significant influence in their decision-making.

### The vulnerabilities of being in a foreign work culture

One consistent theme reported by migrants was feeling oppressed in a new work culture, which was vastly different from home, even though their “*guangong*” (supervisors) were from China. Many described work hierarchy as oppressive, and dependent on “*guanxi*”, the relationship with one’s supervisor. Those who did not meet expectations faced harsh treatment and were transferred to tougher work sites for “*zanghuo*” (dirty work). These unpleasant consequences were summed up as “*diaonan*” (making things difficult). Many migrants reported being willing to “*chiku*”, literally meaning to “eat suffering”, because of the high agent fees. One migrant described his sense of vulnerability, while displaying his resilience:

*Migrant: In China, the supervisors dare not shout at us but here, things are different.*

*Interviewer: Why?*

*Migrant: The China supervisors here have been here long enough. […] They have a lot of authority and decide how much you are paid. […] We’re all on tenterhooks. We are powerless before our supervisors here.*

*Interviewer: How do you overcome your challenges?*

*Migrant: Just persist and let it pass. (M002)*

### 健康是本钱: health is my capital

When asked what “health” meant to them, many migrants used a Chinese proverb “健康是本钱”, referring to health as a form of capital to earn money for their families:

*In China, we have a saying, “*健康是本钱*” (health is my capital). With health, I can work. Without it, I cannot do anything. (M009)*

Most migrants shared that health implied the ability to earn money. An injured migrant described how the lack of health affected his ability to make a living, “*Work will not be the same with my injury. I will earn less with lighter work.” (M010)* Nonetheless, this seemingly materialistic view belies the perspective of most migrants, who believe that money is secondary to health and family.

*Health is of utmost importance as without it I cannot earn money for a better life for my family. (M007)*

Such strong familial ties were common among nearly all migrants. The intensity of this breadwinning responsibility which most migrants feel is well-expressed as such, “*Health is supreme because my family needs me to support them. If I am unwell and my family needs to support me instead, I would be a sinner.” (M013)*

However, a minority of migrants who suffered disproportionately severe injuries had a renewed perspective of health. One migrant with a skull fracture and five operations in total reported a change in perspective before and after his injury. This was one of a few exceptions:

*Interviewer: Why is health important to you?*

*Migrant: I can still see the world, I’m lucky to be alive.*

*Interviewer: Is this a new perspective after your injury?*

*Migrant: Yes. Before I got injured, health was just being able to earn lots of money. (M015)*

Another migrant who fell from an eight-metre height shared how health transcended monetary value:

*Health is important like kinship, money is secondary. It does not matter how much I earn, health is more important. […] Because with health I can be with my family and be happy. (M005)*

In summary, health was perceived as a form of capital and asset, to enable them to work hard to better the lives of their family. However, in the face of unusually severe injuries, participants tended to view health more poignantly, as an opportunity to spend time with their families and savour life.

### Pathways to healthcare access by Chinese migrants in Singapore

**Fig. 1:**
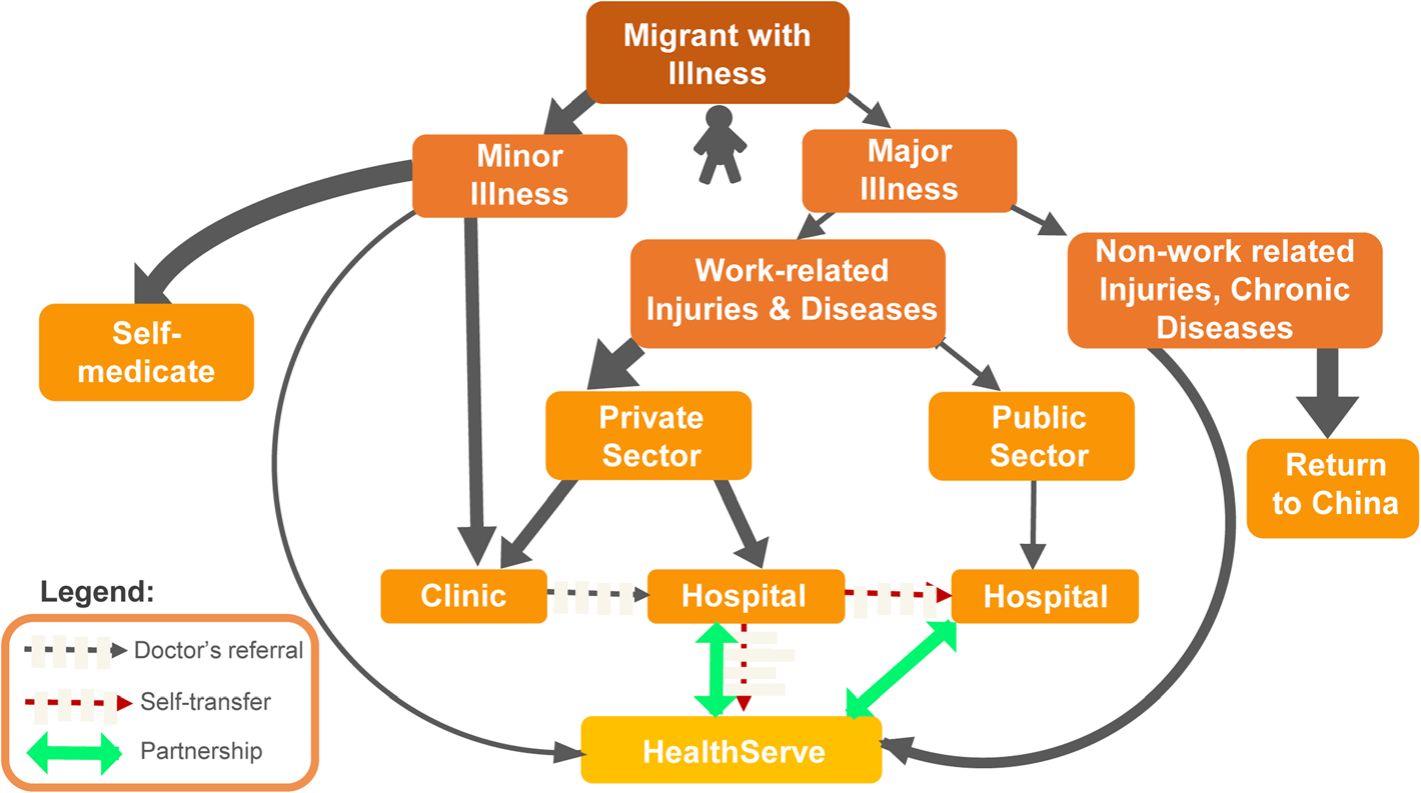
Pathways to healthcare access by Chinese migrants in Singapore

Healthcare access by Chinese migrants in Singapore can be divided into six major pathways, and depends on whether the illness was perceived as minor or major, and whether self-medication, returning to China for treatment, the private or public health sector was chosen (Fig. 1). The thickness of the arrowed lines reflects how common each route is, with greater line thickness representing the more common route reported by more migrants.

For minor illnesses, there were three possible routes: the majority would first self-medicate. If self-medication was unhelpful, they would visit private clinics in or outside their worksite. A minority who heard of HealthServe would visit its clinic. For major illnesses, five routes could be identified. For chronic illness or major non-work related injuries, most participants preferred to return to China. Several interviewees, however, reported being comfortable seeking help from HealthServe.

Work-related injuries and diseases can be covered by the Work Injury Compensation Act (WICA), which allows employees to claim for medical leave wages for the days that the employee was issued with medical leave due to the work injury or disease, medical expenses, and lump sum compensation for permanent incapacity or death [15]. However, participants reported they often did not have the choice of private or public healthcare provider. The hierarchical work culture meant this decision depended on their supervisors, who would usually choose the private sector. The minority with access to the public sector reported significantly less inconvenience obtaining medical documentation, medical leave, and having their work injuries reported for compensation through the Work Injury Compensation Act. However, most migrants interviewed were channelled to private company doctors by their employers and faced challenges in getting their work injuries reported, obtaining medical leave wages, or gaining fair compensation. Due to their strong social network as a cultural continuation of social cohesiveness in China, migrants reported freely offering information on alternative sources of help such as HealthServe, the public healthcare sector, or MOM.

### Perspectives of migrants on minor illnesses

For minor illnesses such as influenza, gastritis, and skin disease, most migrants reported they would self-medicate using medications brought from China, for convenience and to save money. A few stated acquiring medicine in China was more convenient, since medicines were easily available from pharmacies or “*cunyi*”s (village doctors). In contrast, acquiring medication in Singapore was “more troublesome”. Many migrants reported cultural barriers such as inconvenience in finding a clinic, not being able to buy medicines without a doctor’s consultation, and not being able to understand English. Nearly all participants reported that cost was an important consideration, as migrants who take a day off work without producing a Medical Certificate (MC) from a doctor do not receive the day’s wage [16].

While participants reported that larger companies would reimburse workers’ medical fees at private clinics for minor ailments, other smaller companies did not necessarily have the same policy. One migrant stated that even with being paid medical leave wages, it may not cover the medical fees. Due to the high financial investment in agent fees to gain employment overseas, many were willing to suffer and work through minor illnesses, to avoid loss of pay.

*Interviewer: So you were still working with fever?*

*Migrant: Yes. If not, I don’t get paid. I just had to bear with it. (M017)*

Only one migrant from an exceptionally established company expressed gratitude to the generous insurance provided, “*For small illness like flu, we can visit the doctor first, pay and get reimbursed. The company will add the sum to our salary. […] If it is a more serious illness and you don’t have money, the company will send a guarantor’s letter so that you can stay in the hospital first. (M013)*

### Perspectives of migrants on major illnesses

All migrants stated a medical examination was necessary before qualifying for work in Singapore. Hence, most did not know anyone with serious chronic diseases. Because the medical insurance for migrants does not cover non-work related illnesses, those with chronic diseases reported their preference to return to China, as treatment would be more affordable. One migrant shared about his friend’s situation:

*Interviewer: Is your friend with kidney stones cured?*

*Migrant: Yes. The boss asked him to go back to China and get treated. But he came back again after getting cured because the pay is higher here. (M010)*

One staff reported the challenges of uninsured non-work injuries such as cancer:

*There is no provision for illnesses like cancer for which insurance will pay. But for ‘humane medicine’, there needs to be provision for such situations. (S021)*

Migrants who suffered work injuries had mixed experiences. Many were funnelled directly to private practitioners by their employers. They described negative experiences, such as suspected collusion between their employers and doctors, language barriers, and difficulties obtaining medical documents and having their work injuries reported. They shared observations of conflict of interest in the doctor-patient relationships at private hospitals, resulting of mistrust of their doctors:

*The doctors think of the interests of the bosses because the bosses are paying them. Bosses usually have their selected hospitals and will not let you go to the public hospitals. (M014)*

Most HealthServe staff members reported the potential conflict of interest to patients when privatized doctors are contracted by employers, as they may be under pressure to issue less MC by employers [25].

*Staff: The reason for giving two days MC is because if you have three days MC or more, you have to report the incident to MOM. Then the employers will get a penalty.*

Public hospitals were generally perceived to be more equitable and trustworthy. Such information is spread by word-of-mouth among migrants. In Mandarin, they are described as “big hospitals”, paralleling the trust migrants place in bigger city hospitals in China. This is in contrast to the mistrust they have in private hospitals, which are described as “small hospitals”, corresponding to the less advanced, smaller facilities in China. One migrant shared his difficulties in reporting his work injury:

*I should have seen the doctors at the big (public) hospital from the start as it is easier to get my documents there. […] They would report your case after you get your MC. The boss has no authority over them. At the small hospital, I could not even see my MC. (M019)*

One injured migrant who was sent to a private hospital was unable to obtain any medical documents and thus took his friends’ advice to transfer himself to a public hospital, where he could obtain his MCs. This was recounted by a few participants:

*The public hospitals are fairer. They do not rely on “guanxi” (private ties) with employers, unlike private doctors. (M015)*

Nonetheless, one staff highlighted that migrants may prefer to present to General Practitioners over the Emergency Department of public hospitals:

*General Practitioners are cheaper than A&E (Accident & Emergency). Often migrants prefer to pay from their own pocket so they can continue to work without their bosses knowing. (S023)*

### The recovery and repatriation process

One common challenge reported by most participants was the long wait from the point of injury to being repatriated back home. During this time, they usually suffered a loss of income. The mental stress of waiting for compensation is compounded by most participants’ unwillingness to share news of their injury with their family. Many explained why using the Chinese proverb “*baoxibubaoyou”,* which refers to their culture of reporting only good news and hiding bad news. Surprisingly, several migrants, all of whom had suffered work injuries, expressed their desire to return to Singapore to work again:

*Although I sustained work injury here, I would still come again if I can. The laws here are good and the government will help you if the company does not pay. (M004)*

HealthServe staff with experience of visiting repatriated migrants in their villages in China explained the challenges of injured migrants assimilating back home:

*Those who were seriously injured can’t work when they return home and their families suffer more problems. […] Whenever we visit China, we meet with the family and share what the migrant went through. Through this, we salvaged a few marriages. (S024)*

**Fig. 2:**
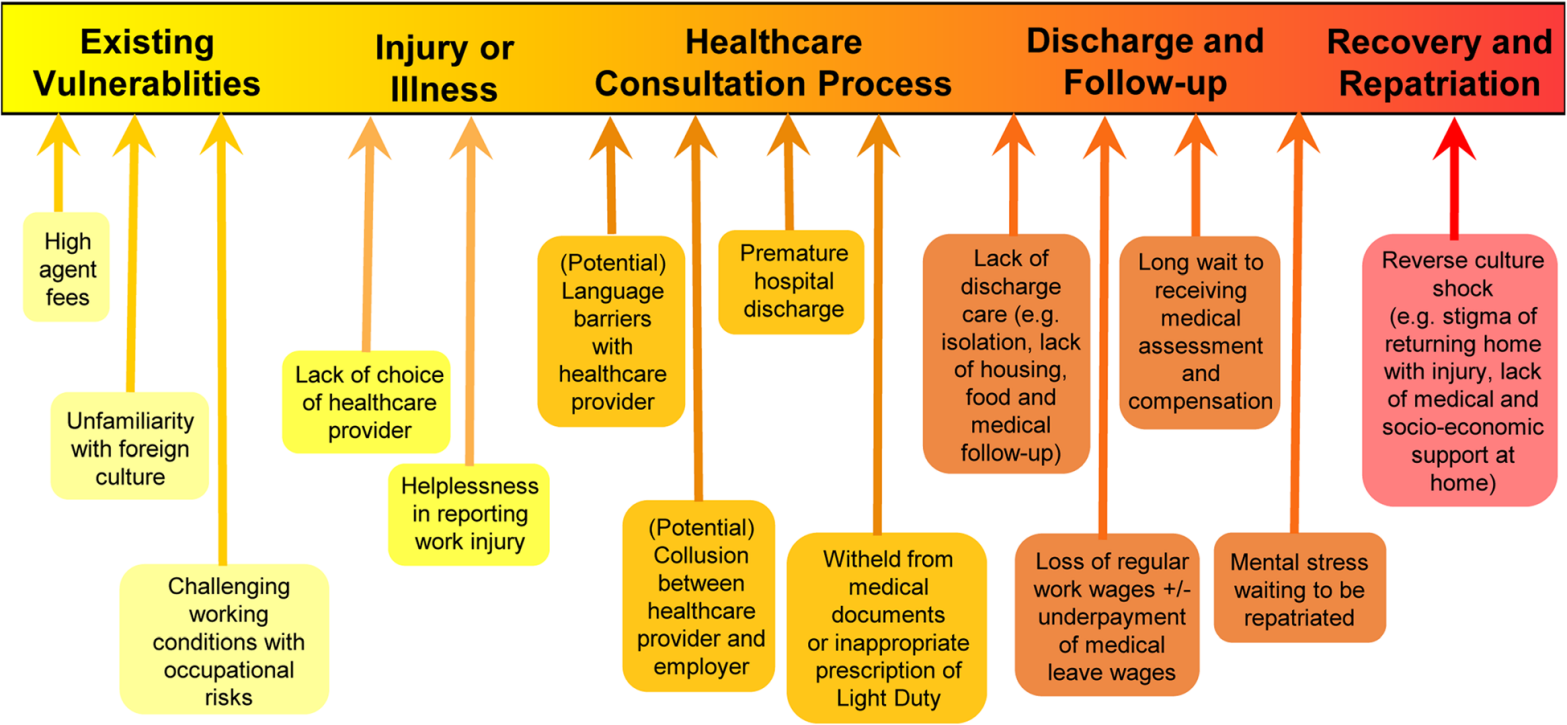
Reported barriers to accessing healthcare services

The story of one of the migrants illustratesthe points of vulnerability in a migrant’s journey (Fig. 2), and the interdependency of barriers and enabling factors for migrants accessing healthcare.

Bing is a 53-year old farmer migrant from Jiangsu, China. Being the sole breadwinner of his family with elderly parents and two sons, his motivation for migrating was to earn more money for his family. Upon persuasion by his friends and agent, he chose Singapore for its reputation of good security. When he first arrived, his expectations were challenged. He stayed in a container with 16 people. The long working hours and pay were different from the rosy picture his agent had painted to him. However, having paid high agent fees of USD$5000, he decided to “*chiku*” (eat bitterness). As time passed, his salary increased from USD$1100 to USD$1500 a month. Bing shared his challenges of working in an unfamiliar country*, “We do not have time to find out where to see a doctor as we work most of the time. It is only when we fall sick that we start sourcing for information.”*

During work, he fell through a scaffold while carrying 25 kilograms worth of cement and suffered a left knee fracture. His supervisor advised him to rest, but due to persistent pain, he walked to a nearby private clinic. The doctor advised him to seek help from a public hospital if the pain persisted. A few days later, on his way to a public hospital nearby, Bing’s manager stopped him and took him to a private hospital situated further away. Bing was unable to choose his healthcare provider of choice and explains this vulnerability that many migrants face*, “When we are injured, we don’t have the power to choose our healthcare provider. When you’re in pain, you’re under the control of your boss. After I went to the public hospital, my relationship with my employer turned sour. My supervisors told me I should not have gone to the public hospital as I did not have their permission.”*

During the healthcare consultation, Bing faced language barriers in communicating with the doctor at the private hospital, who spoke directly to his employer. He describes his challenges*, “I understood when the private doctor and the safety officer spoke in Mandarin but then they switched to English. The doctor said that I needed 4 months rest before I could do light work. Yet he wrote the notice for light duty for my boss instead. He said and did different things. […] I think they “chuantong” (colluded).”*

Bing underwent an operation and was not allowed to keep any medical documents. Bing explains the challenges he faced*, “They had me discharged on the day I had my operation. […] The safety officer said that I was the one who insisted on discharging. My employers had all my MCs so I could not report my work injury. They did not give me my salary for 2 months. I asked the safety officer if mine was considered a work injury but he said no because I was not hospitalized.”*

Bing suffered a loss of income. Without any medical documents, he could not report his work injury or receive his fair compensation. During this time, he suffered much mental stress. Thus, Bing heeded his friends’ advice and presented at the Emergency Department of a public hospital. For the first time, he received his copy of MC which he finally used to report his work injury. Bing shares his sentiments about his private and public sector healthcare experiences:

*Migrant: The public hospitals are more “gongping” (fair). Their service is good.*

*Interviewer: Are there barriers between the doctors and you?*

*Migrant: No barrier. I trust the doctors at the public hospital.*

*Interviewer: But the barrier exists with the private doctors?*

*Migrant: Yes, whatever documents are issued is given to the boss only.*

A year ago, Bing had suffered a skin allergy to cement. When self-medication proved ineffective, he visited a private clinic which cost USD$40. He paid for himself. His friend then recommended him to HealthServe. After his work injury, Bing returned to HealthServe, which provided him with free housing, food, medicine, emotional and spiritual support. When asked to describe his experience at HealthServe, he used a phrase which was commonly used by other migrants, “*xiangjiarenyiyang*”, which means the staff was like family. At the point that this paper was written, Bing was still awaiting part of his compensation from his company, 10 months after his injury. He mentioned he was looking forward to returning to China to start a small business.

## Discussion

This study contributes to the perspectives of mainland Chinese migrants and non-governmental staff and volunteers serving them, on their access to primary, secondary and tertiary care in Singapore. Most Chinese migrants interviewed reported feeling culturally and socially accepted in Singapore, where many speak Mandarin. This is in contrast to the social exclusion reported by mainland Chinese migrants in Hong Kong, which has an intense political rivalry with China [26].

Health was perceived by participants as an asset and an important form of capital, needed to work, earn and contribute to improving the lives of their families. This is supported by another local cross-sectional study of 525 migrant workers from Bangladeshi or Myanmar nationality exploring health-seeking behaviours, where the majority of those who saw a doctor did so because they felt medical care would enable them to work better [4]. The Chinese proverb “健康是本钱” (health is my capital) in our study succinctly encapsulates this sense of ownership over one’s health, for the sake of working to provide for one’s family.

Models have been constructed to explain how health capital differs from other forms of human capital, as one’s stock of health uniquely determines the total time one spends on producing monetary earnings and commodities [27, 28]. Lee’s study revealed that migrant workers with more rest days were significantly more likely to see a doctor when sick, suggesting that the maintenance of health was more likely when it did not affect the production of money [4]. This is consistent with the concept of health expressed by our study participants, as they viewed sick days as a source of disutility, and viewed health as what Grossman describes as an “investment commodity”, which determines the time available for market and non-market activities [27]. Our study findings align with Grossman’s concept of health capital, which argues that an increase in the stock of health reduces the time lost from activities, and the monetary value of this reduction is an index of the return to an investment in health [27].

One of the four basic premises which underlie the theory of the demand for health is reflected among the perspectives of migrants interviewed- that health is desirable but not valued above all else [28]. When probed, participants suggested that health was a stepping stone towards achieving their primary aim, which was to work harder to better the lives of their family, even if they had to endure physical or mental hardships, expressed in term “*chiku*” (eat suffering).

Due to limited understanding of the healthcare landscape in an unfamiliar environment and the strict hierarchical work culture on worksites, most migrants interviewed faced challenges in choosing their preferred healthcare provider. Negative experiences reported while attending the private sector included perceived collusion between doctors and employers, withholding of medical documentation, insufficient hospitalization or medical leave, and lack of discharge care. Difficulties with reporting work injury resulted in a loss of medical leave wages, an unduly long wait to receiving fair medical assessment and compensation, and the mental stress of waiting to be repatriated. The significance of mental stress should not be underestimated, as findings of a study of Chinese migrant workers within China itself suggested that migrant residents had a higher prevalence of depression and less utilization of healthcare services and self-medication [29].

Nonetheless, migrants interviewed often displayed great resourcefulness to tap on their interconnected networks, to look for alternative sources of help such as HealthServe, through word-of-mouth from their friends. This form of social capital can be succinctly described by Coleman as “the value of social norms of reciprocity, social networks, and mutual trust”, which also affects health [30]. In fact, access to health services has been suggested as a pathway by which social capital influences health outcomes [31]. Social capital is thought to affect health outcomes through norm observances, family support, and benefits mediated through extra-familial networks [32]. Interestingly, HealthServe, which is often described as being “like family”, appears to strengthen their social capital through its function as a community of financial, socio-emotional and even spiritual support. An article analysing social networks among rural-urban migrants in China revealed, however, that social networks are a double-edged sword, which can offer assistance but also restrict healthcare access [33].

Within this context the work of Bourdieu is called upon, particularly his concepts of *habitus*, fields, and capital. *Habitus* has been used widely to refer to a system of dispositions through which we perceive, judge and act in the world [34]. Fields are the different arenas or networks in which people reproduce their dispositions [35]. For example, Chinese migrants are now navigating different fields with different rules, and they have developed new networks to access and operate within them. Their habitus or dispositions is shaped by their experiences in particular positions in the social structure [34, 36]. This means that they are shared by people with similar experiences such as nationality or social class [36].

In this context, their ability to navigate the different fields also depends on their particular endowment with capital [36]. Bourdieu distinguishes between four types of capital: social capital, economic capital, cultural capital and symbolic capital [34]. For example, in our study, economic capital refers to the material conditions that migrants have been able to accumulate, which influenced and restricted their choices and decisions. Social capital refers to social networks with family, friends and acquaintances, and voluntary organizations in the different fields as mentioned above. Cultural capital refers to cultural knowledge covering a wide range of resources such as educational credentials, general cultural awareness and communication abilities [34, 37]. Our findings suggest the importance of these concepts to uncover the ways in which migrants’ habitus, their cultural, social and economic capitals have influenced their abilities to access services in unfamiliar fields with a strict hierarchical work culture and with a health system with complex pathways.

With regards to primary care for minor illnesses and other non-work related illnesses or chronic diseases, most of the migrants interviewed indicated they would self-medicate, pay on their own to see a private doctor, or return to China for long-term treatment. This response is supported by Lee’s study, which shows that nearly half of the interview subjects would self-medicate, and in those who saw a doctor, private general practitioners and workplace doctors were the most visited, followed by government clinics and hospitals, and the HealthServe clinic [4]. Nearly all HealthServe staff interviewed highlighted the gap in caring for migrant workers who suffer non-work related illnesses and are vulnerable to being laid off and repatriated by employers who do not wish to bear the added costs of care. These findings support existing findings from literature on health and access to care for migrant workers in Singapore, which suggest that while the majority of migrant workers seek healthcare when needed, many still face barriers in accessing care [4].

Some findings of this study were, however, inconsistent with those of Lee’s and team, who found that migrants who earned lower salaries were significantly more likely to believe they would have to pay for their own healthcare or be uncertain about who would pay [4]. Our findings suggested that Chinese migrants, who earned comparably more per month, would also pay for themselves for minor illnesses at private clinics or prefer to return home to China for treatment of non-work related, chronic diseases, as they were not confident that their company would bear the costs. Of those participants whose employers paid for the medical expenses incurred at their assigned private hospitals, their employers often expressed dissatisfaction when care was transferred to a public hospital. The above supports concerns that more needs to be done to lower the barriers of migrant workers to access healthcare, with medical expenses to be fairly borne by employers at their preferred healthcare provider of choice. It is also noteworthy that migrants expressed trust and recounted overwhelmingly positive experiences in the public healthcare sector, which they shared with other Singaporean citizens. In comparison, a study on health constraints among rural-urban migrants in China reported that migrants experienced discrimination because of their lack of opportunity to share the health services equally with urban residents [38].

“Health is my capital” well encapsulates the sense of ownership and resourcefulness of the migrants interviewed, in their access to healthcare in a foreign land. A study suggests that migration results in new ways of mobilizing, enacting, and validating cultural capital which builds on the power relations and resources of the country of origin and country of migration [39]. This can be seen in the migrants interviewed, as they harness their culturally-proactive attitudes from their homeland to negotiate and navigate new healthcare landscapes. Future research can be done on the various forms of capital which migrants build upon to access healthcare in unfamiliar contexts.

### Limitations and strengths

Inevitably, this study has some limitations. The study was conducted at a single NGO site in Singapore, at HealthServe. Thus, experiences could have been skewed as those with injuries and compensation issues tended to present there. This group is likely a vulnerable group who may report more negative experiences. Experiences of migrants presenting at other NGOs or even directly at their place of work could differ. For example, this data excludes the healthcare perspectives and experiences of migrants who fail to present at all to any healthcare providers.

The study’s strengths include access to Chinese migrants who faced challenges accessing primary, secondary and tertiary care in Singapore. In 2014, HealthServe had 3424 consultations, 216 work injury cases and 226 non-injury cases [40]. Using HealthServe as a setting for our study gave us access to this vulnerable group who faced challenges in their healthcare access, financial situations, and legal issues. Working with HealthServe also gave us access to staff who regularly served migrants in need, and who could thus give us their perspectives on policy recommendations and the way forward.

### Implications for policy and practice

While policies and guidelines such as the Employment Act exist in Singapore to protect migrant workers, gaps emerge in the background of a constant flux of change [13]. The magnitude and frequency of such gaps must be investigated, and policy recommendations to protect vulnerable populations must adapt accordingly. Table 3 provides a summary of key recommendations for improving access to healthcare arising from the analysis of participants’ responses.

**Table 3:** Proposed recommendations for policy and practice

Proposed Recommendations for Policy and Practice	Specific Ways to Implement Recommendations
Ensure open access to transparent and fair healthcare providers	- Designate appointed non-biased, government to attend to migrant workers. Consider setting up specific units in public hospitals which specialize in migrant cases. - Increase migrants’ awareness on their rights to choose their healthcare provider during standard orientation briefing by Ministry of Manpower. Include information on where and how to seek help during illness. - Disengage private sector from employers to prevent conflict of interest. - Regular audits of migrant health records to survey the severity of injuries and corresponding medical leave and work injury compensation outcomes. - Establish government-run clinics in dormitories, which are allowed to perform medical assessments for migrant workers. - Provide a flat rate or state-owned insurance for migrant workers.
Strengthen Partnerships between HealthServe and Multiple Stakeholders	Hospitals: - HealthServe to establish partnerships with private and public hospitals to ensure open communication regarding migrant cases, easy retrieval of medical assessments, medical leave, and reports for compensation, and to ensure patient privacy in healthcare consultation. - Design hospital protocols to identify vulnerable migrants early, for early intervention by migrant NGOs. - Ensure smooth transitions in discharge care, especially in the first month of care after surgery. Other Stakeholders: - Encourage platforms for regular open communication between Ministry of Manpower, dormitory owners, employers, construction unions, schools and other migrant NGOs to improve working and living conditions for migrant workers.
Develop Culturally-Competent Healthcare Policies and an Inclusive Society for Migrants	- Improve training of healthcare providers to increase awareness of migrant health issues, such as their medico-legal issues and compensation, and cultural understanding of migrants. - Ensure translations in various languages to improve health literacy for migrants. - Ensure verbal and/or written translations in communication with migrant patients. - Encourage community building activities between Singaporeans and migrant workers to bridge cultural barriers and build friendships.
Ensure Continuity of Care after Repatriation	- Ensure follow-up with migrant workers who have repatriated, to ensure their smooth assimilation back to their home culture.

Most migrants reported a lack of power to choose their healthcare providers, and a sense of helplessness when their healthcare outcomes were determined by their employers, whom they perceived to collude with their doctors. Disengaging the private sector from employers by designating appointed public hospitals to attend to migrant workers or availing opportunities for migrants to choose their healthcare providers could be potential solutions. Regular audits of medical records of migrant worker patients to survey the severity of injuries and corresponding MC and work injury compensation outcomes could help, especially in the private sector, given potential manpower constraints at public hospitals.

Strengthening partnerships between HealthServe and various stakeholders would facilitate communication between different government and private, medical and non-medical agencies involved in migrant care. To deliver culturally-competent care to migrant patients, training among medical students could include a component on migrant issues. Efforts to nurture an inclusive society for all can be made through the development of culturally-sensitive healthcare policies, such as health services which ensure continuity of care after repatriation. In countries like Britain, attention has been drawn to policymakers to develop culturally-sensitive health services for Chinese immigrants, acknowledging their heterogeneous backgrounds and diverse migration experiences [41].

## Conclusion

Migrants are a vulnerable group in Singapore. While they are able to access healthcare, improvements by multiple stakeholders through enforcement, adjustment and refinement of guidelines are needed to overcome their challenges in a dynamic migration landscape. A concerted effort for open communication between employers, MOM, migrant workers, private and public healthcare sectors and migrant NGOs is needed. Medical staff should adopt culturally-competent relational skills, while employers should adhere to governmental guidelines to protect migrant workers. Additionally, policymakers should examine how migrant workers can gain access to healthcare providers of their choice without pressure from their employers. The evidence provided in this paper suggests that further legislation is needed to protect this vulnerable population, to strengthen their access to healthcare and to help them maximize their health capital for meaningful gains.
